# Effects of Syllable Rate on Neuro-Behavioral Synchronization Across Modalities: Brain Oscillations and Speech Productions

**DOI:** 10.1162/nol_a_00102

**Published:** 2023-05-11

**Authors:** Deling He, Eugene H. Buder, Gavin M. Bidelman

**Affiliations:** School of Communication Sciences & Disorders, University of Memphis, Memphis, TN, USA; Institute for Intelligent Systems, University of Memphis, Memphis, TN, USA; Department of Speech, Language and Hearing Sciences, Indiana University, Bloomington, IN, USA; Program in Neuroscience, Indiana University, Bloomington, IN, USA

**Keywords:** cortical tracking, phase locking, sensorimotor integration, speech rhythm, speech synchronization

## Abstract

Considerable work suggests the dominant syllable rhythm of the acoustic envelope is remarkably similar across languages (∼4–5 Hz) and that oscillatory brain activity tracks these quasiperiodic rhythms to facilitate speech processing. However, whether this fundamental periodicity represents a common organizing principle in both auditory and motor systems involved in speech has not been explicitly tested. To evaluate relations between entrainment in the perceptual and production domains, we measured individuals’ (i) neuroacoustic tracking of the EEG to speech trains and their (ii) simultaneous and non-simultaneous productions synchronized to syllable rates between 2.5 and 8.5 Hz. Productions made without concurrent auditory presentation isolated motor speech functions more purely. We show that neural synchronization flexibly adapts to the heard stimuli in a rate-dependent manner, but that phase locking is boosted near ∼4.5 Hz, the purported dominant rate of speech. Cued speech productions (recruit sensorimotor interaction) were optimal between 2.5 and 4.5 Hz, suggesting a low-frequency constraint on motor output and/or sensorimotor integration. In contrast, “pure” motor productions (without concurrent sound cues) were most precisely generated at rates of 4.5 and 5.5 Hz, paralleling the neuroacoustic data. Correlations further revealed strong links between receptive (EEG) and production synchronization abilities; individuals with stronger auditory-perceptual entrainment better matched speech rhythms motorically. Together, our findings support an intimate link between exogenous and endogenous rhythmic processing that is optimized at 4–5 Hz in both auditory and motor systems. Parallels across modalities could result from dynamics of the speech motor system coupled with experience-dependent tuning of the perceptual system via the sensorimotor interface.

## INTRODUCTION

The auditory cortex faithfully tracks amplitude modulations in continuous sounds, regardless of whether those acoustic events are speech (Ahissar et al., 2001; Casas et al., 2021; Luo & Poeppel, 2007), modulated white noise (Henry & Obleser, 2012), or clicks (Will & Berg, 2007). This phenomenon, whereby a listener’s rhythmic brain activity (i.e., *oscillations*) entrains to the physical signal, is described as *neural synchronization* or *cortical tracking*. Neurocognitive models suggest that the phase of ongoing brain oscillations, especially within the low theta band (4–8 Hz), lock to the slowly varying amplitude envelope to parse continuous sounds into discrete segments necessary for speech comprehension (Doelling et al., 2014; Ghitza, 2011, 2012; Giraud & Poeppel, 2012; Luo & Poeppel, 2007). In particular, speech syllable rhythms, which exhibit a quasiregularity in their envelope modulation (Ding et al., 2017; Tilsen & Johnson, 2008), have been used to study how the brain parses the continuous speech stream (Ghitza, 2012; Hyafil et al., 2015). However, such brain entrainment is not solely low-level neural activity that simply mirrors the acoustic attributes of speech. Rather, entrained responses also serve to facilitate speech comprehension (Doelling et al., 2014; Luo & Poeppel, 2007; Peelle et al., 2013). These studies demonstrate that the degree to which auditory cortical activity tracks acoustic speech (and non-speech) signals provides an important mechanism for perception.

Syllable rhythms in speech range in speed from 2–8 Hz (Ding et al., 2017). With this variability in mind, it is natural to ask whether the brain’s speech systems are equally efficient across syllable rates, or instead are tuned to a specific natural speech rhythm. Indeed, the majority of the world’s languages unfold at rates centered near 4–5 Hz and neuroacoustic entrainment is enhanced at these ecological syllable speeds (Ding et al., 2017; Poeppel & Assaneo, 2020). In their neuroimaging study, Assaneo and Poeppel (2018) demonstrated that auditory entrainment (i.e., sound-to-brain synchronization) is modulated by speech rates from 2.5 to 6.5 Hz but declines at faster rates. In contrast, a more restricted 2.5–4.5 Hz frequency coupling was found in phase-locked responses to speech between auditory and motor cortices (i.e., brain-to-brain synchronization; Assaneo & Poeppel, 2018). This suggests that while neural oscillations can entrain to a wider band of external rhythms (e.g., 2.5–6.5 Hz), motor cortex resonates at select frequencies to emphasize syllable coding at 4.5 Hz. A neural model was proposed accordingly: speech-motor cortical function is modeled as a neural oscillator, an element capable of generating rhythmic activity, with maximal coupling to auditory system at 4.5 Hz. Such studies suggest, at least theoretically, a convergence of the frequency of endogenous brain rhythms during speech production and the cortical encoding of speech at its input.

In parallel with auditory-motor cortex coupling, behavioral sensorimotor synchronization has been extensively characterized by having individuals produce certain movements in time along with external physical events. Sensorimotor skills have most often been studied in the form of tapping to a periodic stimulus (Repp, 2005). The rate limits of synchronization in beat tapping approximately correspond with inter-onset intervals between 100 ms (Pressing & Jolley-Rogers, 1997) and 1800 ms (Miyake et al., 2004; Repp, 2005). However, these examples of non-speech motor synchronization may not generalize to speech considering its unique nature in human cognition. The therapeutic benefits of synchronizing to audio or visual speech productions, referred to speech entrainment, has been demonstrated in patients with Broca’s aphasia (Fridriksson et al., 2012; Thors, 2019). However, experience-based rates (i.e., patient’s most comfortable rate) have been implicitly used in speech entrainment tasks rather than systematically verified. Additionally, using a spontaneous speech synchronization (SSS) task, Assaneo et al. (2019) found some listeners involuntarily match their speech with external rhythm while others remain impervious. Listeners were instructed to freely produce syllable trains while hearing syllables at rates of 4.5 syll/s with the goal of monitoring the occurrence of syllables. Their data established a link between word learning capabilities and sensorimotor speech synchrony. Critically, the optimal rate of the speech sounds in those studies was assumed to be close to the natural/normal speaking rate (i.e., ∼4–5 Hz). Uncertainty also persists regarding how wider ranges of syllable rates might affect speech synchronization. Further, studies have shown that better rhythm perception abilities are indicative of increased conversational quality mediated by better speech entrainment (Wynn et al., 2022). Thus, it is highly plausible that an individual’s preference for certain stimulus rates perceptually might facilitate their successfully entrainment at similar preferred rates during production. To address this knowledge gap and explicitly test for frequency-specific coupling in speech perception and production, sensorimotor and auditory synchronization must be measured in a common paradigm.

In the present study, we aimed to empirically compare syllable rate sensitivity of the auditory-perceptual and (sensori)motor systems. In doing so, we ask whether brain and speech entrainment is or is not selectively tuned to the fundamental periodicity inherent to speech (∼4.5 Hz) and thus represents a common organizing principle of processing across modalities. This notion has been suggested, but to our knowledge has remain largely untested, in prominent neurocognitive models of speech processing (Assaneo et al., 2021; Assaneo & Poeppel, 2018; Poeppel & Assaneo, 2020). To this end, we measured neuroacoustic tracking of listeners’ electroencephalogram (EEG) to speech syllable trains to quantify their perceptual entrainment to speech. To quantify motor entrainment, we measured speech productions where participants synchronized to a wide range of syllable rates between 2.5 and 8.5 Hz along with (simultaneous production) or without (non-simultaneous production) a concurrent auditory speech stimulus. Employing both production tasks allowed us to isolate more or less pure measures of motor system by including/excluding external auditory stimuli. Brain-behavior correlations and comparisons of rate profiles across EEG and production data allowed us to explicitly characterize possible links between auditory neural and motor production entrainment mechanisms of speech processing.

## MATERIALS AND METHODS

### Participants

Fifteen young adults participated in the study (mean age 26.7 ± 3.4 years; 10/5 females/males). (One additional participant completed the experiment but their data were lost due to a logging error). They were of mixed race and ethnicity. Ten were native English speakers and five were bilingual with English as a second language. Several participants had musical training (mean 9.9 ± 3.8 years). All participants were right-handed (Oldfield, 1971) and reported no history of neuropsychiatric disorders. All had normal hearing sensitivity, defined as air-conduction pure tone thresholds ≤ 25 dB HL (hearing level) at octave frequencies from 500 Hz to 4000 Hz. Listeners were provided written informed consent in compliance with a protocol approved by the University of Memphis institutional review board and were monetarily compensated for their time.

### Stimuli

#### EEG stimuli

We used stimuli inspired by Assaneo and Poeppel (2018) to characterize brain synchrony to rhythmic speech. Each consisted of trains of a single repeating syllable from the set /ba/, /ma/, /wa/, /va/ (random draw). Individual tokens were synthesized from online text-to-speech software (FromTextToSpeech.com, n.d.) using a male voice, and time compressed in Praat to 120 ms durations (Boersma & Weenink, 2013). Tokens were concatenated to create syllable trains of 6 s duration. To vary syllable rate, we parametrically varied the silent gap between tokens from 0 to 280 ms to create seven continuous streams of speech syllables with rates of 2.5, 3.5, 4.5, 5.5, 6.5, 7.5, and 8.5 syll/s. In practice, the 8.5 Hz condition was presented at a nominal rate of 8.33 Hz to achieve the fastest presentation speed possible given the 120 ms duration of our individual speech tokens.

#### Speech production stimuli

To assess simultaneous (cued) and non-simultaneous (un-cued) speech production synchronization, we generated another two sets of stimuli adapted from the SSS task (Assaneo et al., 2019). To study the non-simultaneous rhythm production, we used syllable trains of continuous repetitions of /ta/ lasting for 10 s.

For simultaneous rhythm production, we used 60 s long syllable streams with 16 distinct syllables (unique consonant-vowel combinations) that were randomly concatenated. We generated seven rate conditions (∼2.5–8.5 syll/s). This was achieved by temporally compressing/expanding the 4.5 Hz syllable stream from Assaneo et al. (2019) by the appropriate scale factor using the “Lengthen” algorithm in Praat (Boersma & Weenink, 2013).

### Data Acquisition and Preprocessing

Participants were seated comfortably in front of a PC monitor and completed the three experimental tasks in a double-walled, sound-attenuating booth (Industrial Acoustics Company, 2023). Auditory stimuli were presented binaurally at 82 dB SPL (sound pressure level) via electromagnetically shielded ER-2 insert earphones (Etymotic, 2023). Stimuli and task instructions were controlled by MATLAB 2013 (MathWorks, 2013) routed to a TDT RP2 signal processing interface (Tucker-Davis Technologies, 2022). Speech production samples were recorded digitally with a professional microphone (Blue Yeti USB, Logitech; 44100 Hz; 16 bits; cardioid pattern; Blue Yeti, 2022).

#### EEG data

**Figure 1.**
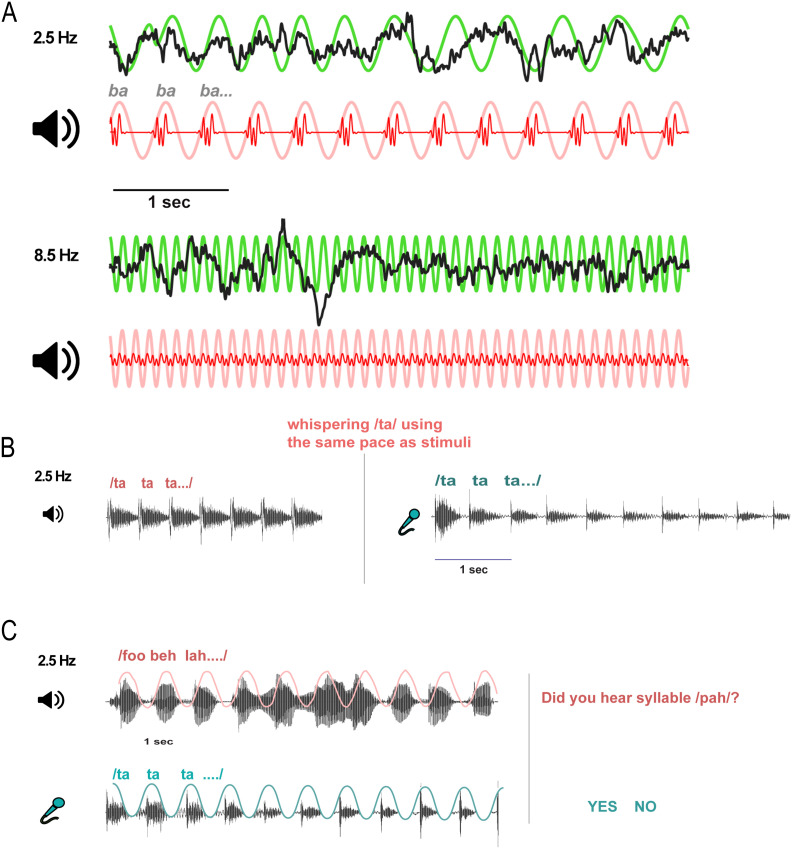
Examples of neural entrainment and speech synchronizations. (A) Brain entrainment to speech envelope for a slower (2.5 syll/s) and higher (8.5 syll/s) syllable rate. Black = cortical EEG responses; green = schematized EEG envelope; red = stimulus waveform; pink = speech fundamental envelope. (B) Schematic of the non-simultaneous (un-cued) speech production task (2.5 Hz rate). (C) Schematic of the cued (simultaneous) production synchronization task (2.5 Hz rate). Pink = auditory stimuli; light blue = speech production samples.

During neural recordings, participants listened to rhythmic syllable trains (Figure 1A). To maintain attention, they were instructed to identify which syllable (i.e., /ba/, /ma/, /wa/, /va/) was presented at the end of the trial via button press. There was no time constraint to respond, and the next trial started after the button press. Listeners heard 10 trials of each 6 s syllable train per syllable rate condition. Rate and syllable token were randomized within and between participants.

Continuous EEGs were recorded differentially between Ag/AgCl disc electrodes placed on the scalp at the mid-hairline referenced to linked mastoids (A1/A2) (mid-forehead = ground). This single channel, sparse montage is highly effective for recording auditory cortical EEG given their fronto-central scalp topography (Bidelman et al., 2013; Picton et al., 1999). Interelectrode impedance was kept ≤ 10 kΩ. EEGs were digitized at 1000 Hz uisngSynAmps RT amplifiers (Compumedics Neuroscan, 2022) and an online passband of 0–400 Hz. Neural signals were bandpass filtered (0.9–30 Hz; 10th order Butterworth), epoched into individual 6 s trial segments synchronized to the audio stimuli, and concatenated. This resulted in 60 s of EEG data per rate condition. Eyeblinks were then nullified in the continuous EEG via a wavelet-based denoising algorithm (Khatun et al., 2016). Trials were averaged in the time domain to derive cortical neural oscillation for each condition. We measured synchronization between brain and acoustic speech signals via phase-locking values (PLV; see Phase-Locking Value).

#### Speech production data

##### Non-simultaneous syllable rhythm synchronization (Figure 1B).

Participants first listened to rhythmic syllable trains (/ta/ repeated for 10 s). They were instructed to then whisper /ta/ with the same pace as the previous stimulus for 10 s (i.e., without a concurrent audio stimulus). With this explicit instruction and whispering articulation requirement, we aimed to investigate intentional speech rhythm production guided by internal rhythmic cues, minimizing self-auditory feedback. The procedure was repeated twice for each rate condition. Two runs were conducted in anticipation of avoiding possible practice effects. However, data from the two runs were highly correlated (*r*_2.5_ = 0.75, *r*_3.5_ = 0.88, *r*_4.5_ = 0.80, *r*_5.5_ = 0.91, *r*_6.5_ = 0.86, *r*_7.5_ = 0.77, *r*_8.5_ = 0.82, *p* < 0.001), indicating good test-retest repeatability. Moreover, paired *t* tests further confirmed the two runs did not differ at any of the rates (*p*_2.5_ = 0.85, *p*_3.5_ = 0.66, *p*_4.5_ = 0.22, *p*_5.5_ = 0.17, *p*_6.5_ = 0.23, *p*_7.5_ = 0.94, *p*_8.5_ = 0.17).

##### Simultaneous syllable rhythm synchronization (Figure 1C).

We adapted the SSS test (Assaneo et al., 2019) to measure cued motor speech to auditory synchronization. Participants were instructed to continuously whisper /ta/ while *concurrently* listening to a rhythmic syllable stream for 60 s. By employing whisper and insert earphones, we aimed to avoid participants’ using their own production sounds as auditory feedback to their speech output. After each trial, listeners indicated whether a target syllable were presented in the previous stream. Four target syllables were randomly chosen from a pool of eight (50% were from the syllable stream). Importantly, we did not explicitly instruct participants to synchronize to the external audio rhythm and we also removed their training session. In previous studies using the SSS, listeners first heard a fixed syllable rate at 4.5 Hz presented auditorily (Assaneo et al., 2019). This may have primed them to produce syllables with the same pace leading to an artificial increase in performance at 4.5 Hz. Participants were informed the goal was to correctly identify the target syllable and that the speech they heard was only to increase task difficulty. The purpose of this behavioral task was to prevent participants from intentionally matching their speech to the aural inputs by directing their attention to the syllable identification task.

### Data Analysis: Quantifying Synchronization and Rate Accuracy

We performed analyses using custom scripts written in MATLAB and used TF32 software to examine the rate of acoustic signals (Milenkovic, 2002).

#### Phase-locking value

We measured brain-to-stimulus synchronization (and similarly speech-to-stimulus synchronization) as a function of frequency via PLV; Lachaux et al., 1999). Neural and auditory signals were bandpass filtered (±0.5 Hz) around each frequency bin from 1 to 12 Hz (0.5 Hz steps). The envelope was calculated as the absolute value of the signal’s Hilbert transform. PLV was then computed in each narrow frequency band according to Equation 1.

$$PLV=\frac{1}{T}\left|\sum_{t=1}^{T}e^{i\left[\theta_{1}\left(t\right)-\theta_{2}\left(t\right)\right]}\right|$$ (1)

where *θ*_1_(*t*) and *θ*_2_(*t*) are the Hilbert phases of the EEG and stimulus signals, respectively. Intuitively, PLV describes the consistency in phase difference (and by reciprocal, the correspondence) between the two signals over time. PLV ranges from 0–1, where 0 represents no (random) phase synchrony and 1 reflects perfect phase synchrony between signals. The PLV was computed for windows of 6 s length and averaged within each rate condition. Repeating this procedure across frequencies (1–12 Hz; 0.5 Hz steps) resulted in a continuous function of PLV describing the degree of brain-to-speech synchronization across the bandwidth of interest (e.g., Assaneo et al., 2019). PLVs were then baselined in the frequency domain by centering each function on 0 by subtracting the value of the first (i.e., 1 Hz) frequency bin. This allowed us to evaluate the relative change in stimulus-evoked PLV above the noise floor of the metric. We then measured the peak magnitude from each PLV function to trace changes in brain-to-speech synchronization with increasing syllable rate.

For speech production-to-stimulus synchronization (which are both acoustic signals), we processed the recordings using the speech modulation procedure described by Tilsen and Johnson (2008). We first discarded the first/last 5 s of each recording to avoid onset/offset artifacts and then normalized the amplitude. We then bandpass filtered the signal (3000–4000 Hz; 4th order Butterworth) to highlight the voiceless whispered energy followed by half-wave rectification to extract the speech envelope. We then lowpass filtered (f_c_ = 30 Hz), downsampled (F_s_ = 80 Hz), windowed (Tukey window), and de-meaned the envelope modulated signal to isolate slower speech rhythms. As in the brain-to-stimulus synchronization analysis, we then measured PLV between the acoustic productions and speech stimulus for each rate.

#### Speech rate

As an alternate approach to corroborate the automatic rate measures, we manually counted syllables for each 10 s recording of participants’ non-simultaneous productions from wideband spectrograms computed in TF32. Speech rate was calculated as the number of syllables per s; onset and offset silences were not included in these calculations. Since the audio recordings of implicit speech rate productions were 60 s each, we further validated the reliability of syllable counting by applying an automatic peak finding algorithm. Again, the first/last 5 s were discarded to avoid transient onset/offset effects. We then extracted the Hilbert envelope and smoothed the signal using a 30 ms moving average. The amplitude was normalized before and after envelope extraction. Lastly, we employed MATLAB’s ‘findpeaks’ function (minpeakhight = 0.08, minpeakprominence = 0.01, minpeakdistance = 117ms) to automatically detect and measure syllable peaks. Visual inspection and auditory playback were used to determine these optimal parameters. The speech rate calculated from the spectrogram and peak finding algorithm were highly correlated (*r* = 0.95; *p* < 0.0001) confirming the reliability of the automatic analysis approach.

### Statistical Analysis

Unless noted otherwise, we analyzed the data using one-way, mixed-model analyses of variance (ANOVAs) in R (Version 1.3.1073; ‘lme4’ package; Bates et al., 2015) with rate (7 levels; 2.5–8.5 Hz) as a categorical fixed effect and subjects as random factor (e.g., PLV ∼ rate + (1|subject)) to assess whether the brain-to-stimulus and speech-to-stimulus synchrony differed across syllable rate. The Tukey post hoc test for multiple comparisons was used. Moreover, to test whether PLV at 4.5 Hz is enhanced, following the omnibus ANOVA, we used an a priori contrast to compare neural PLV at 4.5 Hz versus other syllable rates. For production data, we tested whether participants’ produced rate achieved the target syllable rate using one-sample Shapiro *t* test and Wilcox signed rank test for the simultaneous (implicit) and non-simultaneous (explicit) rate production tasks, respectively. Significance in these tests indicates participant’s production speed deviated from (e.g., was slower/faster than) the nominal stimulus rate. To assess brain-behavior associations, we first used Pearson’s correlations to test the across individual association after aggregating across rates between neural and production PLV. We then used repeated measures correlations (rmCorr; Bakdash & Marusich, 2017) to assess within-subject relations between neural and acoustic synchrony measures. Unlike conventional correlations, rmCorr accounts for non-independence among each listener’s observations and measures within-subject correlations by evaluating the common intra-individual association between two measures. Initial diagnostics (quantile–quantile plot and residual plots) were used to verify normality and homogeneity assumptions. Consequently, PLV measures were square-root transformed to allow for parametric ANOVAs. Behavioral data from the EEG task (i.e., percentage of correctly perceived syllables) were rationalized arcsine transformed (Studebaker, 1985). A priori significance level was set at *α* = 0.05. Effect sizes are reported as $n_{p}^{2}$.

## RESULTS

### Cortical Oscillation Synchrony Is Enhanced at ∼4.5 Hz Syllable Rate

**Figure 2.**
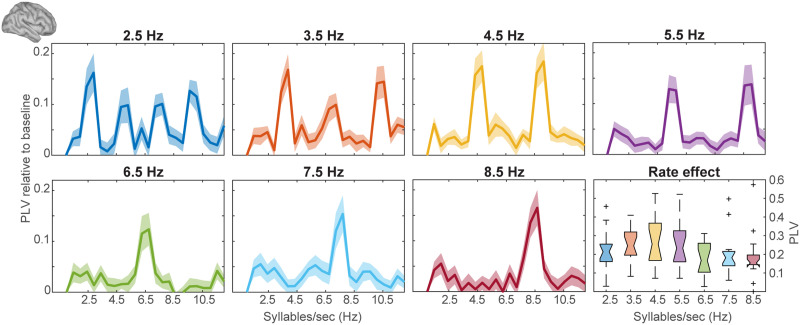
Phase-locked neural oscillations synchronize to the rate of the syllable envelope. The phase-locked value (PLV) increment from baseline between neuroelectric activities and the stimuli envelope across frequency are enhanced at 4.5 Hz. Note the peak in the PLV close to the nominal syllable rate as well as higher harmonics. Similar harmonics were observed in the spectra of the acoustic stimulus envelopes, owing to the non-sinusoidal nature of speech waveforms. The bottom right panel represents the distribution of peak PLV across participants as a function of stimulus syllable rate. Shading = ±1 standard error of the mean.

The percentage of correctly perceived syllables during EEG recordings showed no significant difference (*F*_6,90_ = 1.76, *p* = 0.1162, $n_{p}^{2}$ = 0.11) across conditions, confirming participants were equally engaged in the listening task across rates. We evaluated neural-speech PLV (Figure 2) to assess how ongoing brain activity synchronized to speech (Assaneo & Poeppel, 2018) over an expanded range of ecologically valid syllable rates (2.5–8.5 Hz) characteristic of most languages (Ding et al., 2017; Poeppel & Assaneo, 2020). Each PLV plot shows a strong peak at the fundamental frequency surrounding the rate of the stimulus as well as additional peaks at harmonic frequencies. Harmonic energy was also present in the acoustic stimuli. An ANOVA conducted on neural PLV revealed a main effect of syllable rate (*F*_6,90_ = 3.76, *p* = 0.0022, $n_{p}^{2}$ = 0.2). An a priori contrast showed that PLV was stronger for 4.5 Hz compared to all other rates (*p* = 0.026). Interestingly, 4.5 Hz corresponds with the mean syllable rate in English (Goswami & Leong, 2013; Greenberg et al., 2003) as well as most other languages (Ding et al., 2017; Varnet et al., 2017). Our results reinforce the notion that neural oscillations synchronize to the speech envelope and are modulated by syllable rate. More critically, we observed an enhancement of PLV at the frequency close to the predominant syllable rhythm (4.5 syll/s) inherent to most languages, suggesting a preferred rate of neural oscillation coherent with listeners’ long-term listening experience.

### Spontaneous Speech Synchronization Is Restricted to Slower Rates

**Figure 3.**
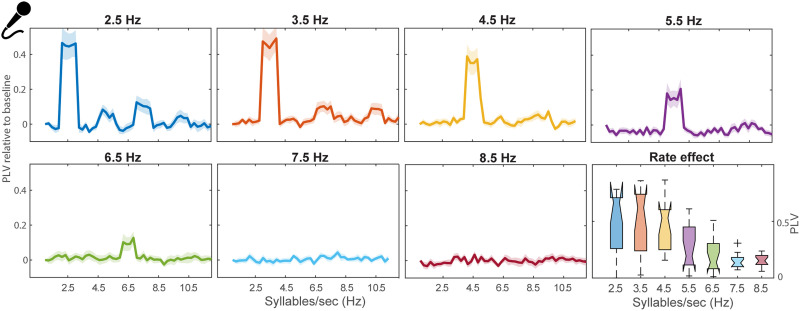
Simultaneous speech synchronization to syllable trains is modulated by rate. The phase-locking value (PLV) increment against baseline was computed between acoustic stimuli and listeners’ speech productions. Note the performance optimizes at slower (2.5–4.5 Hz) compared with higher rates (5.5–8.5 Hz). The bottom right panel represents the distribution of peak PLV across participants as a function of stimulus syllable rate. Shading = ±1 standard error of the mean.

We next examined whether listeners’ cued speech productions were synchronized to the simultaneous audio track at various syllable rates (Figure 3). Speech-to-stimulus PLVs showed selective peaks at the audio speech rhythm that declined with increasing rate above ∼6.5 Hz (main effect of syllable rate: *F*_6,90_ = 14.355, *p* < 0.0001, $n_{p}^{2}$ = 0.49). Post hoc analysis revealed stronger PLV for slower (2.5–4.5 Hz) versus faster (5.5–8.5 Hz) rates (all *p* values < 0.05). These results suggest that participants can only synchronize their speech productions to relatively slow syllable rate (i.e., motor performance is akin to a lowpass filter).

### Correspondence Between Syllable Perception and Production

**Figure 4.**
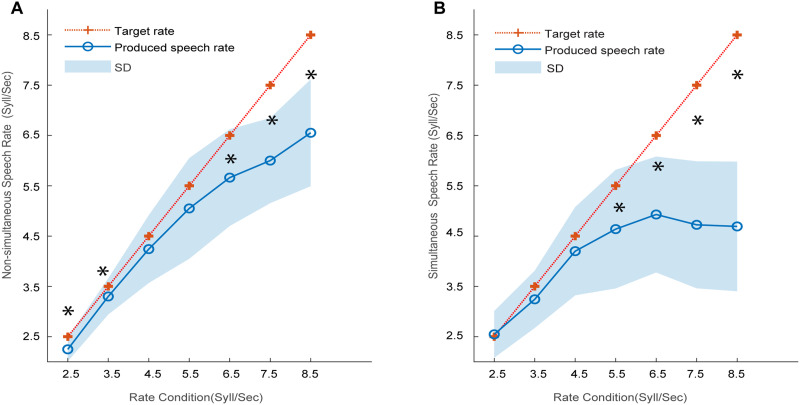
Participants’ produced speech rate compared to the target rate of auditory stimuli. (A) Speech rate was produced *after* rhythmic syllable trains were presented (non-simultaneous) with explicit instructions of pace duplication. (B) Participants produced syllables while *simultaneously* listening to rhythmic streams with implicit rate synchronization. *p < 0.05, significant deviations from the expected rate (red +) based on one-sample tests against the nominal (target) rate value. Shaded region = ±1 standard deviation (SD).

To explore the link between syllable rhythm entrainment in perception and production, we measured participants’ accuracy for producing target syllables under the two experimental settings: one following an explicit instruction to replicate a previously heard rhythm (*non-simultaneous*/*un-cued* productions) and the other with implicit instruction to mirror a concurrently presented syllable train (*simultaneous*/*cued* production). One sample *t* tests showed that for non-simultaneously produced syllable rate (NSR; Figure 4A), participants only hit target rates at 4.5 and 5.5 syll/s (4.5 Hz: *t*(14) = −1.49, *p* = 0.16; 5.5 Hz: *t*(14) = −1.74, *p* = 0.10). However, the variability in productions also appeared to differ across rates. Indeed, measuring the mean absolute deviation of responses, we found smaller variability in productions at rates of 2.5 and 3.5 Hz versus 4.5 and 5.5 Hz (*p* = 0.003, one-way ANOVA). This suggests at least part of the effect at 4.5–5.5 Hz in Figure 4A might be attributed to more/less precise productions across rates. Notably, productions deviated from (were slower than) the target speeds above 6.5 Hz indicating they failed to keep pace with the audio stimulus. Simultaneously produced rate (SSR; Figure 4B) measures showed highly accurate reproductions for ∼2.5–4.5 Hz (*p*_2.5_ = 0.46, *p*_3.5_ = 0.13, *p*_4.5_ = 0.26), with slowing of production at higher rates. The results of SSR were consistent with the enhanced speech-to-stimulus PLV at 2.5–4.5 Hz (see Figure 3).

### Brain-Behavior Correlations Between Production and Neural Speech Entrainment Accuracy

**Figure 5.**
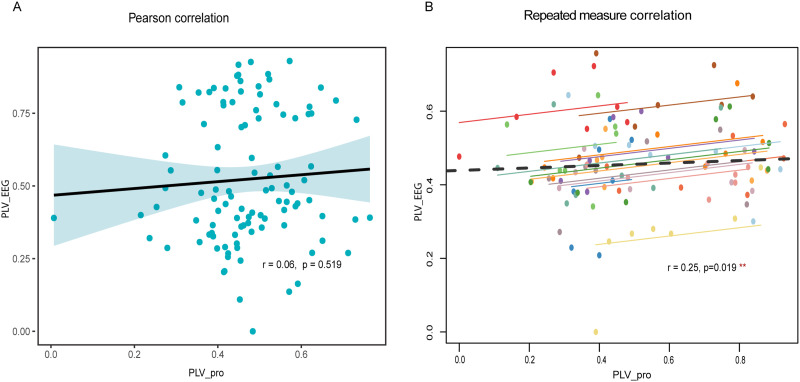
Correlations between brain and production synchronization to speech. (A) Pearson correlation (between-subjects) aggregating across rate conditions between neural and production PLV. (B) Repeated measures correlations (within-subjects) between neural and production PLV. PLV_EEG = neural-to-stimulus PLV; PLV_pro = speech-to-stimulus PLV. Dots = individual participants’ responses; solid lines = within-subject fits to each individual’s data across the seven rates; dashed line = linear fit across the aggregate sample. *p < 0.05, **p < 0.01, ***p < 0.001.

To explore the relationship between auditory and motor (production) responses, we conducted between- and within-subject correlations. Figure 5A suggests a non-significant relation between neural and production PLV when the data are considered on the whole, without respect to each individual. Indeed, rmCorr correlations assessing within-subject correspondence revealed a positive correlation between neural and speech PLV (*r* = 0.25, *p* = 0.019, Figure 5B), indicating an auditory-motor relation in rhythmic synchronization abilities at the individual level.

## DISCUSSION

By measuring EEG oscillations and acoustical speech productions in response to syllable trains presented at various rates, the current study evaluated syllable rate-dependencies in auditory neural entrainment and simultaneous speech synchronization, and possible dynamic relations between these domains. We first confirmed that auditory brain activity robustly synchronizes to the ongoing speech envelope and flexibly adapts to the speed of syllable trains in a rate-dependent manner (Assaneo & Poeppel, 2018; Ding et al., 2016; Rimmele et al., 2021; Will & Berg, 2007). More interestingly, we found that neuroacoustic phase locking was boosted at rates of ∼4.5 Hz, corresponding to the putative dominant syllable rate observed across languages (Ding et al., 2017). Production data showed that simultaneous speech synchronization to audio rhythms was largely restricted to slower syllable rates (2.5–4.5 Hz). In contrast, and converging with neural data, we found “pure” motor rate productions were produced more accurately; participants more precisely matched syllable rates between 4–5 syll/s even without concurrent auditory cuing. Lastly, correlations between brain and production PLV data extend prior work (Assaneo et al., 2019; Assaneo & Poeppel, 2018) by explicitly linking auditory and motor entrainment skills. We found that individuals with superior auditory entrainment to speech also show enhanced motor speech capabilities in speech-audio synchronization.

### Cortical Oscillation Synchrony Is Modulated by the Heard Syllable Rates

Corroborating previous magnetoencephalography (MEG)/EEG studies (Assaneo & Poeppel, 2018; Ding et al., 2016; Keitel et al., 2018; Teng et al., 2017), our data reveal that low frequency neural oscillatory signals (2.5–8.5 Hz) robustly phase lock and closely mirror the rate of auditorily presented speech. Neuroacoustic phase locking did diminish with increasing rate, consistent with previous findings showing cortical activity fails to synchronize with the envelope of accelerated speech (Ahissar et al., 2001; Nourski et al., 2009). However, entrainment remained above the noise floor even for the fastest syllable rate (8.5 Hz). Accurate neural entrainment to a larger range of frequencies, even some of which are well beyond the regular speeds of intelligible speech (Adams & Moore, 2009; Momtaz et al., 2021; Viemeister, 1979), is perhaps not surprising given the ease at which the auditory system tags temporal acoustic landmarks of speech and non-speech signals (Doelling et al., 2014; Luo & Ding, 2020; Momtaz et al., 2021; Viemeister, 1979). In order to cope with the varying timescales of temporal patterns in speech, neuronal processing must demonstrate rate flexibility (Saltzman & Munhall, 1989; van Lieshout, 2004). Indeed, neural entrainment to external rhythmicity helps ensure proper signal detection (Besle et al., 2011; Stefanics et al., 2010) and facilitates speech comprehension (Doelling et al., 2014; Giraud & Poeppel, 2012; Luo & Poeppel, 2007). One hypothesis of these phenomena is that continuous speech is discretized and segmented on multiscale temporal analysis windows formed by cortical oscillation locking to the input speech rhythm (Ghitza, 2011, 2012, 2014; Giraud & Poeppel, 2012). Our data support these general notions that low-frequency activity of auditory cortex flexibly tracks the speed of the speech envelope via phase synchronization of cortical activity.

Interestingly, cortical responses also showed enhanced phase locking for speech rates proximal to 4.5 Hz. Notably, we observed a bell-shaped rate-dependence with the maximum gain in neural phase locking near 4.5 Hz, which aligns with the dominant spectral profile of syllable rates across languages (Ding et al., 2017). This finding suggests that neural excitability is adjusted to align the acoustic temporal structure of speech such that neural oscillations are tuned to track the acoustic proclivities of natural languages. This is probably coherent with listeners’ long-term listening and speaking experience with the dominant speech rhythms in their language. This supports the notion that neural oscillations coding speech reflect an interplay of input processing and output generation in which the associated neural activities are shaped over time by the statistical structure of speech (Poeppel, 2003).

### Simultaneous Speech-Audio Synchronization Is Rate Restricted

Paralleling our brain-audio synchronization data, we further asked whether simultaneous speech-audio synchronization is affected by syllable rates from 2.5–8.5 syll/s. Importantly, we did not explicitly instruct participants to match the audio rate nor did we provide practice on the task, which we speculate can lead to priming effects and apparent enhancements in synchronization at certain rates (cf. Assaneo et al., 2019). The resulting production data demonstrate that participants’ rhythmic speech output does not uniformly synchronize across rates but is instead severely restricted to slower frequencies from 2.5 to 4.5 Hz. Because the simultaneous production task implicitly instructed listeners to align their self-speech production to heard audio, it necessarily evoked sensorimotor integration. The fact such productions are limited to low rates is consistent with neuroimaging results indicating selective coupling between auditory and motor cortices between 2.5 and 4.5 Hz (Assaneo & Poeppel, 2018). Moreover, the lack of entrainment at higher frequencies as observed in our EEG data perhaps suggests the sensorimotor effects of producing while also listening to speech might create a mixture of entrained brain processes that interfere with or are at least distinct from one another. The shift to slower rate preferences in motor speech synchronization also seems reasonable given the risk of articulatory undershooting when speaking fast (Gay et al., 1974), and the fact that speed of articulation is constrained by the biomechanical limits of articulators. Alternatively, this rate-constriction could result from the oscillator tuning of the motor system in which it involuntarily entrains to (i.e., resonates with) the auditory stimuli when rates are close to its intrinsic rhythm. It is conceivable that auditory-motor interaction has adapted its sensitivity to both forms of natural constraints imposed by the articulatory and motor systems.

Neurophysiologically, this lowpass filter shape could also result if motor responses are dominated by lower-frequency rhythms of the brain. Indeed, delta (0.5–4 Hz) oscillations are thought to reflect endogenous rhythms from primary motor cortex (Keitel & Gross, 2016; Morillon et al., 2019), which can emerge in the absence of acoustic stimulation (Ding et al., 2016; Rimmele et al., 2021). Other possible explanations could be due to the cognitive demands of this task, which consumes heavier cognitive load (Zhou et al., 2018) and requires extra neurocomputational time to match the motor program with the auditory input. Higher task demands would tend to result in successful synchronization only at the easiest (slowest) rate conditions. Low-frequency components of the EEG have been linked to cognitive operations such as sustained attention and working memory (Bidelman et al., 2021; Kirmizi-Alsan et al., 2006). However, this explanation seems speculative since we could not explicitly measure brain oscillations during the production tasks. Instead, the lowpass nature of the simultaneous production data seems parsimoniously described in terms of limits to sensorimotor processing, with more severe constraints imposed by the motor component.

### Non-Simultaneous Productions Highlight an Intrinsic Rhythm at 4–5 Hz

Under an oscillatory framework, different aspects of spoken communication arise from neural oscillations that are accessible for both perception and production. Such oscillations could emerge in the context of input processing and output generation and result in the associated auditory and motor activities that would reflect the structure of speech (Giraud et al., 2007; Giraud & Poeppel, 2012; Liberman & Whalen, 2000).

A second aspect of our study design examined natural speech rate productions via non-simultaneous productions. Some conditions were quite challenging given the rapid production speeds required of the task. This paradigm provided listeners with minimal auditory feedback and thus, better isolated more pure motor system responses during speech output. Without concurrent auditory feedback either from their own speech or external stimuli, possible interference confounds from sound-evoked auditory oscillations mentioned earlier are minimized. Surprisingly, we found participants’ productions under these conditions hit target speeds (statistically speaking) only for rates of 4.5 and 5.5 syll/s. Productions failed to meet targets (i.e., were slower than the nominal rates) at all lower and higher syllable speeds. However, we also note production variability differed as speeds increased (Figure 4A). While we interpret the non-simultaneous data to reflect motor speech function during limited auditory involvement, an alternate interpretation might be the more explicit instruction of rate imitation. Nevertheless, those findings align with our EEG results on auditory entrainment, which similarly showed maximum synchronization at 4.5 Hz and flexibility with wide range of speech rates. This frequency specialization in both the speech perception and production data is suggestive of a resonance of intrinsic neural oscillations representing syllable rhythm (Assaneo & Poeppel, 2018; Luo & Poeppel, 2007; Poeppel & Assaneo, 2020).

The notion of an intrinsic 4–5 Hz rhythm receives further support from several other observations: the predominant peak in speech envelope spectra for many languages and speaking conditions (Ding et al., 2017; Goswami & Leong, 2013); the mean syllable duration in English (∼200 ms; Greenberg et al., 2003; Pellegrino et al., 2011); the coordinated articulation or motor gesture trajectory in sound production (Poeppel & Assaneo, 2020); movement of the lips, tongue, and hyoid with a 5 Hz rhythm during lip-smacking in monkey (Ghazanfar et al., 2012). Neurologically, continuous speech is processed through a temporal integration window of ∼200 ms (period of 4–5 Hz; Luo & Poeppel, 2007). Studies using transcranial alternating current stimulation further show that 5 Hz stimulation enhances cortical entrainment and results in better sentence comprehension (Wilsch et al., 2018). The striking coherence between these divergent methodologies, along with the present data, supports the notion of an intrinsic rhythm at ∼4–5 Hz, a computational primitive in cortical speech processing that also seems to link input and output processing.

### Differences and Limitations to Related Studies

Our stimulus paradigm was adapted from previous neuroimaging studies on neural entrainment to speech rhythm (e.g., Assaneo et al., 2019; Assaneo & Poeppel, 2018). However, there are several distinct aspects of the findings presented here. First, our cortical tracking data observed a stronger brain-to-speech phase synchronization at 4.5 syllables/sec which contrasts with previous reports suggesting auditory cortex is invariant in syllable tracking across rates (Assaneo & Poeppel, 2018). Although listening to rhythmic sounds induces motor cortex (Bengtsson et al., 2009; Wilson et al., 2004), our single channel EEG recordings do not allow us to localize our effects to auditory versus motor cortex generators, per se*.* In this regard, high-density neural recordings (Assaneo & Poeppel, 2018) revealed enhanced intracranial coupling of speech-evoked oscillations between auditory and motor cortices specifically at 4.5 Hz. It is possible then that the gain in cortical phase locking at 4.5 Hz observed in our data reflects neural entrainment in motor-related regions (Assaneo & Poeppel, 2018). Accordingly, other neuroimaging studies have shown that oscillation power in motor areas modulates auditory cortex tracking of acoustic dynamics to facilitate comprehension (Keitel et al., 2017, 2018). Given that the scalp EEG reflects a mixture of intracranial sources, the effects we observe here probably reflect a mixture of entrained oscillations in auditory and motor cortex as suggested by previous MEG studies (Bengtsson et al., 2009; Wilson et al., 2004). Multichannel EEG recordings with source reconstruction analysis could test this hypothesis in future studies. Privileged recruitment of motor brain regions induced by concurrent auditory entrainment may account for the local enhancements in PLV we observe near 4.5 Hz in both our EEG and production data.

Second, we observed a more complex syllable rate-constrained pattern in speech-audio responses (simultaneous productions) but a preferred syllable rhythm for isolated motor synchronization (non-simultaneous productions). To our knowledge, these novel findings have not been observed previously and are only revealed by comparing speech productions with varying degrees of sensory and motor involvement. By explicitly examining multiple modes of production and tasks which tease apart sensory from motor processes, our data establish a link between exogenous and endogenous speech entrainment mechanisms and further reveal unique specialization at 4–5 Hz in both the auditory and motor modalities. These parallel effects likely trace back to the long-term experience of the listener and dominant syllable rates for input processing and output production. In contrast, with concurrent auditory inputs, the rate-restricted pattern could emerge from the tuning of motor oscillator and its interaction with the sensory system. Future studies are also needed to test whether this oscillator tuning is mediated by the better versus worse synchronization performance. It is possible the bimodal distribution in speech-rate synchronization observed in prior work (Assaneo et al., 2019) is apparent only with a very large number of participants or with those with more heterogeneous backgrounds.

In conclusion, our data establish a positive speech perception-production link for rate synchronization. Both perceptual and motor entrainment for speech processing seem optimized for rates between 4 and 5 Hz, the putative nominal speech rate across languages. Still, these links are only identifiable when carefully considering the nature of speech production and tasks that isolate motor from sensorimotor processes. Moreover, we find synchronization skills are subject to individual differences, with performance in the perceptual domain predicting skills in motor domain and vice versa. As such, our findings provide support for theoretical notions of an oscillation-based account of speech processing which organizes both input and output domains of speech processing.

## ACKNOWLEDGMENTS

The authors thank Dr. Bashir Morshed for supplying code for the denoising algorithm, and Dr. M. Florencia Assaneo and Dr. David Poeppel for providing the code for the spontaneous speech synchronization test.

## FUNDING INFORMATION

Gavin M. Bidelman, National Institute on Deafness and Other Communication Disorders (https://dx.doi.org/10.13039/100000055), Award ID: R01DC016267.

## AUTHOR CONTRIBUTIONS

**Deling He**: Conceptualization; Formal analysis; Investigation; Methodology; Visualization; Writing – original draft; Writing – review & editing. **Gavin M. Bidelman**: Conceptualization; Formal analysis; Funding acquisition; Methodology; Supervision; Writing – original draft; Writing – review & editing. **Eugene H. Buder**: Conceptualization; Writing – review & editing.

## DATA AVAILABILITY

The data that support the findings of this study are available on request from the Gavin M. Bidelman (gbidel@indiana.edu). The data are not publicly available because of privacy/ethical restrictions.
